# Impacts of stress on growth and reproductive development of beef heifers

**DOI:** 10.1093/af/vfaf013

**Published:** 2025-08-06

**Authors:** Kelsey M Harvey, Reinaldo Fernandes Cooke, Brooklyn L Laubinger

**Affiliations:** Prairie Research Unit, Mississippi State University, Prairie, MS, 39756; Department of Animal Science, Texas A&M University, College Station, TX, 77843; Prairie Research Unit, Mississippi State University, Prairie, MS, 39756

**Keywords:** beef heifers, exercise, puberty, stocking density, stress, temperament

**Implications**

- Beef heifers with excitable temperament exhibit reduced growth rates and delayed puberty attainment.
- Beef heifers that are acclimated to human handling have accelerated reproductive development and conceive earlier in the breeding season.
- Rearing beef heifers in drylots with a high stocking density stimulates chronic stress and reduces physical activity, resulting in delayed puberty attainment.
- Exposing replacement beef heifers to moderate exercise regimen partially alleviates the detrimental effects of elevated stocking density on puberty attainment.

## Introduction

The success of cow-calf operations is fundamentally tied to the performance and management of beef females, as they are expected to maintain estrous cyclicity and produce one calf per cow annually. A combination of factors, including nutrition and management practices, affect reproductive success in cattle, and therefore overall fertility and herd performance. Replacement heifer development is a cornerstone the cow-calf sector, as these young females offer a valuable opportunity to introduce new genetics to advance herd productivity and profitability. The value of a replacement heifer lies not only in her genetics but also in the management practices employed during her development postweaning. Properly developing replacement heifers involves optimizing nutrition, health, and management to ensure they reach puberty in a timely manner. Proper development of young females before they reach puberty supports herd sustainability and prevent financial losses, as reproductive inefficiency accounts for the culling of approximately one third of a typical cowherd (USDA, 2020).

Stress plays a significant role in heifer development, as physiological and psychological stressors can disrupt biological processes that influence growth, health, and reproductive function (Dobson and Smith, 2000). Stress can present in a variety of forms, including poor handling, disease, nutritional deficiencies, or housing management, leading to elevated circulating cortisol (Carroll and Forsberg, 2007). Prolonged exposure to cortisol can impair immune function, alter metabolic processes, and interfere with development of the reproductive system (Dobson and Smith, 2000), ultimately delaying puberty and affecting fertility and heifer longevity ( Cushman et al., 2013). Stress also negatively impacts cattle growth performance, leading to delayed attainment of necessary body size and composition which are key factors for puberty attainment and reproductive success (Perry, 2016). Identifying specific management strategies to minimize stress for developing replacement heifers represents an opportunity to improve reproductive efficiency, ensuring long-term productivity and sustainability of cow-calf operations. Therefore, the purpose of this review is to highlight research examining the effects of stress during replacement beef heifer development, and subsequent impacts on their reproductive success.

## Stress and Reproduction

In order to be efficient and maximize their reproductive potential, heifers must reach puberty by 12 mo of age, have high conception rates to first breeding by 13 to 15 mo of age, and calve at 24 mo of age (Perry, 2016). Heifers that calve later during their first breeding season face reduced postpartum intervals before the next breeding season, which results in poor conception rates and lighter offspring weaning body weight (**BW**; Cushman et al., 2013). As a result, heifers that reach puberty at an earlier age not only contribute to improved herd fertility but also offer greater economic returns, making replacement heifer development a key driver of both reproductive efficiency and profitability of cow-calf systems (Perry, 2016).

Stress impacts a variety of necessary functions including reproduction and the timing of puberty in heifers, as it interferes with the normal functioning of the hypothalamic–pituitary–gonadal axis (Dobson and Smith, 2000). Exposure to acute stress, such as road transport, disrupts normal pulsatile patterns of gonadotropin releasing hormone (**GnRH**) release and consequently frequency and amplitude of luteinizing hormone (**LH**) pulses are reduced, inhibiting normal ovarian function (Dobson and Smith, 2000). Accordingly, Noble et al. (2000) demonstrated reduced estradiol secretion and delayed or failed ovulation in cows subjected to multiple administrations of adrenocorticotropic hormone (**ACTH**) to mimic chronic stress. In ovariectomized cows exposed to short immobilization to induce acute stress, circulating progesterone concentrations increased following stress (Hollenstein et al., 2006), which was attributed to progesterone synthesis from extra gonadal sources such as the adrenal glands. These results indicate that elevated cortisol and progesterone in response to stress acts in a negative feedback manner on both the hypothalamus and/or pituitary gland, resulting in suboptimal fertility. Estradiol administration to ovariectomized cows exposed to acute stress mitigated the reduction in LH secretion compared to nonstressed cohorts, suggesting estradiol may attenuate stress hormone responses (Hollenstein et al., 2006). However, substantial heterogeneity exists within the literature describing these effects, largely attributed to differences in stress magnitude and estradiol dose (Rhodes et al., 2003). Further research is warranted investigating the extent to which estradiol acts on the hypothalamus, pituitary gland, and the adrenal gland in response to chronic or acute stress.

Most of the literature investigating stress and reproduction in cattle focuses on heat stress and reports suboptimal fertility in females exposed to heat stress during critical times of the estrous cycle (Fernandez-Novo et al., 2020). These outcomes are attributed to impaired gonadotropin secretion, oocyte competence, and embryonic growth in cattle exposed to heat stress (Wolfenson and Roth, 2019). Research in beef cattle demonstrates reduction in pregnancy rates in beef cattle exposed to heat stress during the breeding season (Amundson et al., 2006). Additionally, it is well established that dairy heifers born to dams exposed to heat stress during gestation have compromised growth rates, puberty attainment, and milk production as primiparous cows (Dado-Senn et al., 2020; Laporta et al., 2020; Davidson et al., 2025). However, much of the research investigating heat stress and reproduction has been conducted in dairy cattle, whereas the impact on beef cattle reproductive efficiency deserves further investigation.

## Heifer Temperament

Temperament in beef cattle is defined as the fear-related behavioral responses when exposed to human handling. Temperament traits can include behavior such as responsiveness to handling, reaction to novel stimuli, and flight zone behavior. Typically, cattle that exhibit a more excitable temperament have an aggressive or fearful reaction to human handling procedures (Cooke, 2014). Temperament can be influenced by a variety of factors such as sex, age, handling management, and breed type. Additionally, excitable temperament negatively impacts growth, immune responses, and reproductive success (Cooke, 2014). Circulating cortisol concentrations are heightened in cattle with excitable temperament, which may contribute to altered physiological functions (Cooke, 2014). Mature cows with excitable temperament have decreased pregnancy rate compared to those classified as having adequate temperament in (Cooke et al., 2011, 2012, 2017; Kasimanickam et al., 2014a). Similarly, heifers with adequate temperament have greater BW gain, accelerated puberty attainment (Cooke et al., 2019), and greater pregnancy rates compared with excitable heifers (Kasimanickam et al., 2014b; Dias et al., 2022). Kasimanickam et al. (2014b) demonstrated heifers classified as excitable at the time of artificial insemination had increased circulating cortisol, progesterone, and prolactin during synchronization compared with adequate heifers. These authors concluded that increased circulating concentrations of the aforementioned hormones interfered with GnRH administration at the time of AI, and hence failure of an adequate LH surge to induce ovulation. Assessing temperament traits allows producers to make management decisions to improve the safety of cattle and handlers, ultimately improving overall herd health and longevity.

## Acclimation to Handling

**Figure 1.**
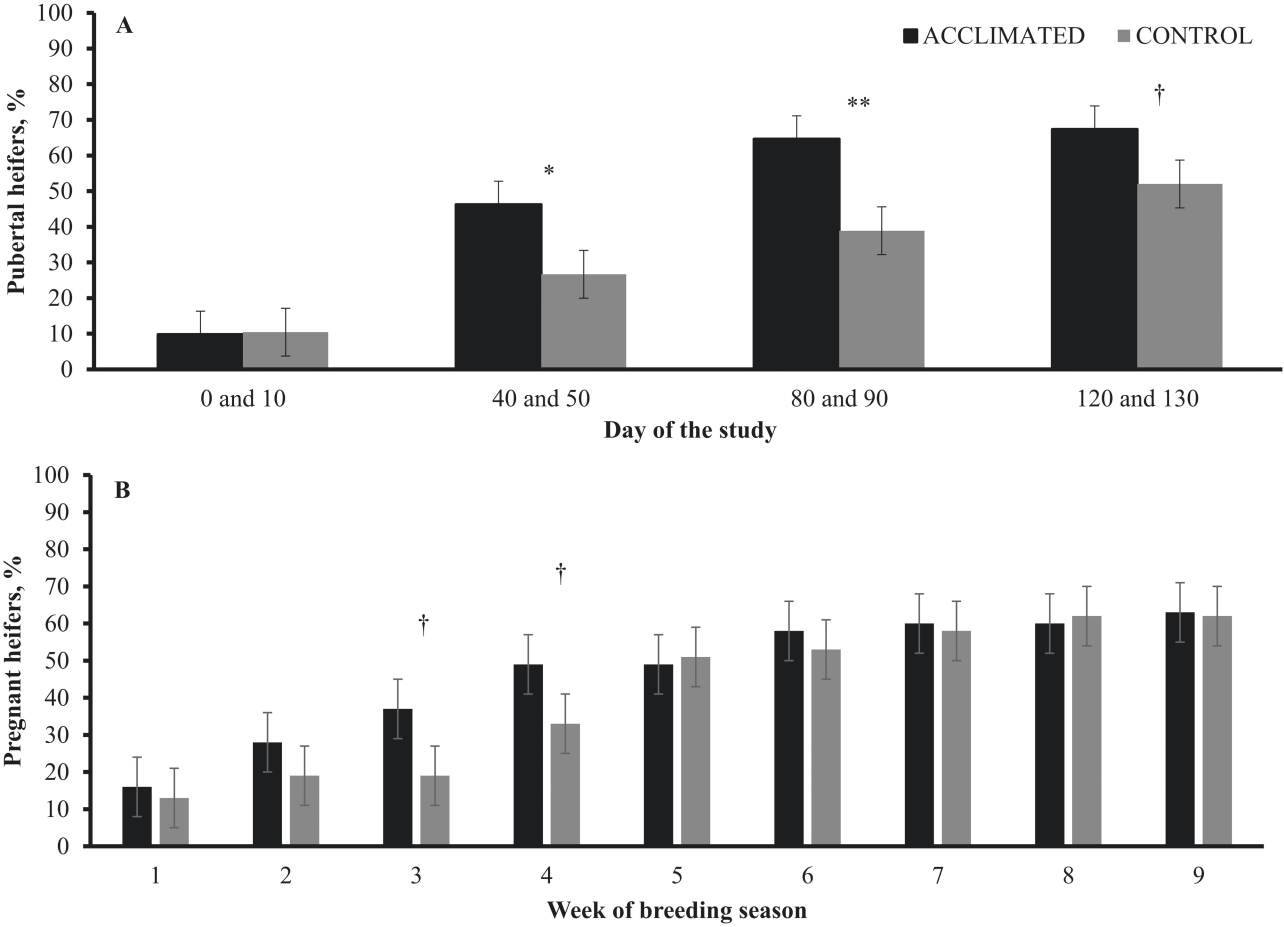
Puberty (Panel A) and pregnancy attainment (Panel B) in *Bos indicus *× *B. taurus* beef heifers exposed or not (**CONTROL**) to handling acclimation procedures (**ACCLIMATED**). Adapted from Cooke et al. (2009), and heifers averaged 269 days age on day 0 of the experiment, and 400 days of age at the beginning of the breeding season. Treatment × day interactions were detected in both panels (*P *≤ 0.04). Within sampling dates: **P < 0.01, *P ≤ 0.05, †*P* = 0.10.

Improving cattle temperament through selective breeding is a long-term process; hence, alternative strategies to mitigate the detrimental effects of excitable temperament in the short term are warranted. Given that frequent handling positively impacts temperament of young cattle, Cooke et al. (2009) sought to investigate the impact of acclimation to human handling on growth and reproductive performance of Brahman-influenced heifers. Heifers assigned to the acclimation procedure were exposed to handling 3 times weekly for 4 wk after weaning, which was applied by processing heifers through a cattle handling facility, whereas control heifers remained undisturbed on pasture. These authors reported that average daily gain (**ADG**), plasma cortisol concentrations, and chute score were reduced in acclimated heifers compared with control cohorts. Heifers exposed to the acclimation procedure had accelerated puberty attainment and conceived earlier in the breeding season compared with nonacclimated heifers (Figure 1).

**Figure 2.**
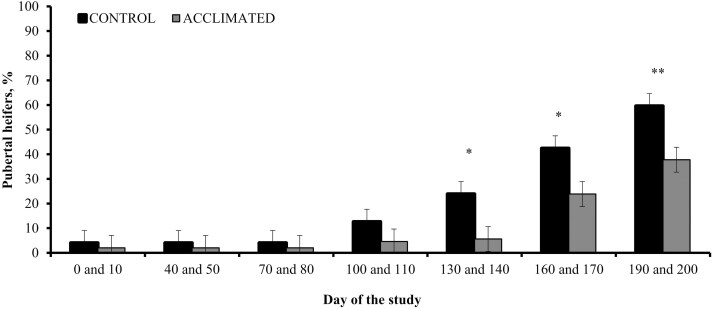
Puberty attainment in *Bos taurus* beef heifers exposed or not (**CONTROL**) to handling acclimation procedures (**ACCLIMATED**). Adapted from Cooke et al. (2012), and heifers averaged 206 days age on day 0. A treatment × day interaction was detected (*P *= 0.01). Within sampling dates: **P < 0.01, *P ≤ 0.05, †*P* = 0.10.

Given that substantial differences exist in reproductive physiology between *Bos indicus* and *B. taurus* heifers (Sartori et al., 2016), Cooke et al. (2012) aimed to investigate the impact of a similar acclimation procedure on growth and puberty attainment of Angus-influenced heifers. These authors also reported reduced mean plasma cortisol in acclimated heifers, whereas no differences in ADG were found. Chute exit velocity, which is a measurement of temperament, was reduced in acclimated compared with control heifers 160 d after the acclimation procedure had concluded. Puberty attainment was accelerated in acclimated heifers, resulting in a greater number of pubertal heifers at breeding compared with control cohorts (Figure 2). In both studies (Cooke et al., 2009, 2012), authors speculated acclimated heifers had reduced circulating cortisol compared with control heifers on a daily basis, as both groups of heifers were routinely exposed to brief human interaction through feeding and traffic at the research unit. Dias et al. (2022) reported that hair cortisol concentration, a biomarker of chronic stress in cattle, decreased from initiation to completion of a 9-d estrus synchronization + artificial insemination (**AI**) protocol, further suggesting that exposing heifers to frequent handling lessens their stress response. Collectively, acclimation procedures may offer a solution to improved the reproductive efficiency of beef heifers, complementing graduate improvements achieved through temperament and genetic selection.

## Stocking Density

The extensive nature of typical cow-calf operations is characterized by land-based management systems in which cattle are primarily housed on pastures (average ≥ 1.0 ha/cow; Asem-Hiablie et al., 2016). However, the availability of grazing lands is increasingly becoming limited due to urban sprawl, environmental challenges, and conversion to crop lands. Many livestock operations are being forced to adopt more intensive management systems, including rearing cattle in confinement that allows for tailored feeding programs to support adequate growth during development. However, this requires careful consideration of space allowance per animal, or stocking density, as high stocking density and associated chronic stress directly influences heifer growth, health, and welfare (Grandin, 2014).

High stocking density and the associated chronic stress (Grandin, 2014) can directly compromise the productivity and reproductive function of beef females (Dobson and Smith, 2000). Mulliniks et al. (2013) demonstrated that heifers reared in drylots had greater BW gain but reduced conception to first breeding compared with cohorts reared on pasture. Petersen et al. (2014) also investigated the impact of rearing heifers in a drylot on activity and indicators of energy metabolism. These authors reported heifers reared in drylots had increased heart rate and were restless compared with heifers housed on pasture. They concluded heifers reared in a high stocking density (11 m^2^/heifer) had a loss of fitness, were less adaptable to winter weather, and experienced greater stress compared with those on pasture despite gaining more BW (Petersen et al., 2014). Both Petersen et al. (2014) and Mulliniks et al. (2013), however, exposed drylot and pasture heifers to different nutritional management that contributed to their reproductive and welfare responses. Perry et al. (2015) investigated the impact of moving drylot developed heifers to pasture after breeding on heifer activity, growth, and pregnancy rate. Heifers were developed in a drylot, and either maintained in the drylot or moved to pasture to graze spring forage 45 d prior to AI. Moving heifers from a drylot to pasture resulted in elevated physical activity and decreased ADG in the initial weeks on pasture due to adaptation to a novel environment, and also reduced pregnancy rate to AI compared to pasture-acclimated heifers (Perry et al., 2015).

**Figure 3.**
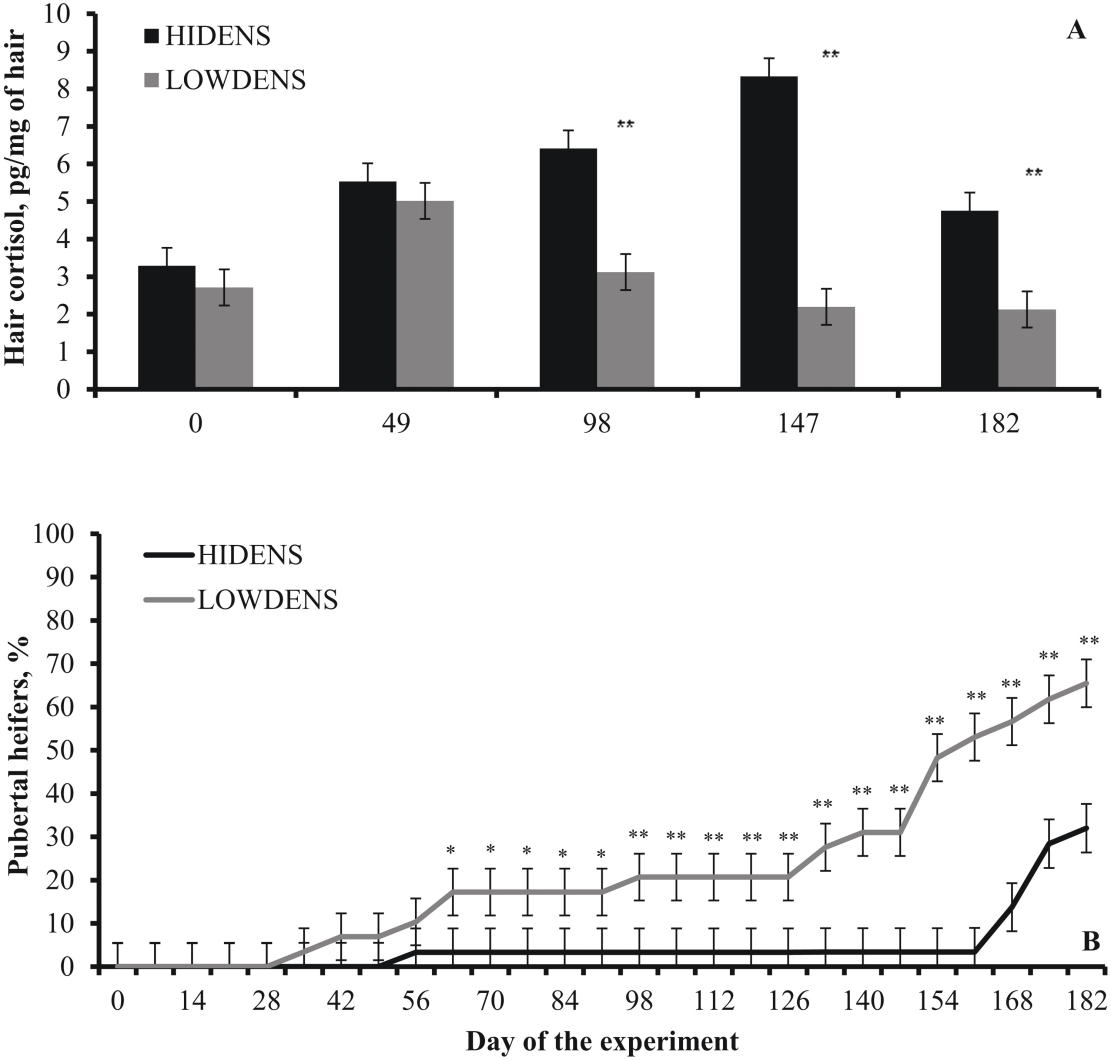
Hair cortisol concentrations (Panel A) and puberty attainment (Panel B) of replacement beef heifers reared in low stocking density (25,000 m^2^/heifer, **LOWDENS**) or high stocking density (14 m^2^/heifer; **HIDENS**). Adapted from Schubach et al. (2017), and heifers averaged 210 days age on day 0. Treatment × day (or week) interactions were detected in both panels (*P* < 0.01). Within sampling dates: * *P* ≤ 0.05 and ** *P* ≤ 0.01.

Based on these findings, our group investigated the impact of stocking density on growth, physical activity, stress-related responses, and puberty attainment of replacement beef heifers (Schubach et al., 2017). This study was conducted with beef heifers reared in drylot pens at a high stocking density (14 m^2^/heifer; **HIDENS**) or on pasture at a low stocking density (25,000 m^2^/heifer; **LOWDENS**) from weaning until their first breeding season. Pastures were harvested for hay prior to winter, resulting in negligible forage available for LOWDENS heifers; therefore, all heifers received the same limit-fed diet. Schubach et al. (2017) reported no differences in ADG between treatments, whereas physical activity measured by steps per week was 6× greater for LOWDENS compared with HIDENS heifers. Hair cortisol concentrations were greater in HIDENS heifers beginning on day 98 compared with LOWDENS cohorts (Figure 3), whereas puberty attainment was delayed in HIDENS heifers (Figure 3). At the end of the experiment (day 182), 31.9% and 65.4% of HIDENS and LOWDENS heifers, respectively, were considered pubertal. Heifer body size and composition play a critical role in reproductive maturation, as sufficient growth and development of appropriate muscle and fat stores are required for the initiation of endocrine activities involved with puberty (Perry, 2016). Despite similar ADG, the HIDENS heifers were older (by 32 d) and heavier (by 48 kg) at puberty attainment compared with LOWDENS heifers.

The novel results from Schubach et al. (2017) indicated that HIDENS heifers experienced chronic stress reactions that delayed their puberty attainment compared with LOWDENS cohorts. Cattle reared in confinement experience restricted physical activity (Perry et al., 2015; Schubach et al., 2017), which can negatively affect their cardiovascular fitness (Petersen et al., 2014) and ability to engage in natural behaviors including grazing (Perry et al., 2015). Accordingly, Schubach et al. (2017) recognized that the specific impacts of restricted physical activity and those of confinement stress in HIDENS heifers were confounded, and indicated the need for further research in this area.

## Exercise as a Strategy to Alleviate Chronic Confinement Stress

Adequate physical activity, specifically through the use of exercise or structured and repetitive physical activity, has direct implications for animal welfare and productivity (Caspersen et al., 1985). Substantial research has investigated the effects of exercise regimen on physiology and welfare of dairy cattle, yet research is limited in beef species. More specifically, increased physical activity or access to an exercise area daily in dairy cattle resulted in decreased cortisol concentration in milk (Veissier et al., 2008), improved overall health (Popescu et al., 2013), and increased frequency of normal social and investigative behavior (Loberg et al., 2004). In feedlot cattle, moderate exercise regimen improved feed efficiency (Daigle et al., 2017) and handling ease (Dunston-Clarke et al., 2020). Additionally, moderate exercise regimen has been shown to improve reproductive efficiency in cattle. Lamb et al. (1979) reported that Holstein heifers exercised for 1.6 km/day for 5 d/week required fewer services per conception and had shorter calving intervals compared with nonexercised cohorts. Corroborating this rationale, research from our group reported benefits of moderate exercise regimen on heifer reproductive development. Cooke et al. (2009) and Cooke et al. (2012) aimed to improve heifer temperament through acclimation to human handling by gathering heifers from pasture and bringing them to the cattle handling facility which was located 2 and 0.6 km from the pasture in Cooke et al. (2009) and Cooke et al. (2012), respectively. These authors inadvertently provided a moderate exercise regimen, and reported acclimated heifers had hastened puberty attainment compared with cohorts that remained on pasture. Collectively, these studies with dairy and beef females suggested a positive effect of moderate exercise on their welfare and reproductive development. Perhaps providing access to an exercise area mitigates the delayed puberty attainment of replacement heifers reared in drylots (Schubach et al., 2017 ). Hence, the next step was to evaluate the effects of a moderate exercise regimen on growth, physical activity, stress-related responses, and puberty attainment of heifers reared in drylots at a high stocking density.

Harvey et al. (2024) evaluated beef heifers reared on pasture at a low stocking density (**CON**; 2,000 m^2^/heifer), drylot pens without access to an exercise area (**DENS**; 14 m^2^/heifer), or in drylot pens with access to an exercise area (**DENS-EX**; 14 m^2^/heifer) from weaning until their first breeding season. More specifically, DENS-EX heifers were given access to an exercise area (30 × 150 m narrow, unpaved, open lane) with no forage available for grazing three times weekly for 60 min each session. Similarly to Schubach et al. (2017), pastures were harvested for hay and mowed as necessary to ensure all heifers received the same limit-fed diet. Accordingly, no treatment differences were detected for heifer ADG during the study. As designed, CON heifers had the greatest physical activity measured by steps per week (23,973 steps/wk), followed by DENS-EX (12,354 steps/wk), whereas DENS heifers had the least physical activity (6,706 steps/wk). While pedometers quantify locomotor activity by measuring steps, the majority of locomotor activity in pasture housed animals can be linked to grazing activity and distance traveled (Kilgour, 2012). Harvey et al. (2024) did not report total distance traveled by heifers but assessed activity parameters using an ear tag accelerometer; Heifers assigned to CON spent more time eating and resting compared with DENS and DENS-EX cohorts. Although pastures were mowed to ensure negligible forage was available, it is likely that CON heifers spent more time searching for grass that was categorized as time spent eating.

**Figure 4.**
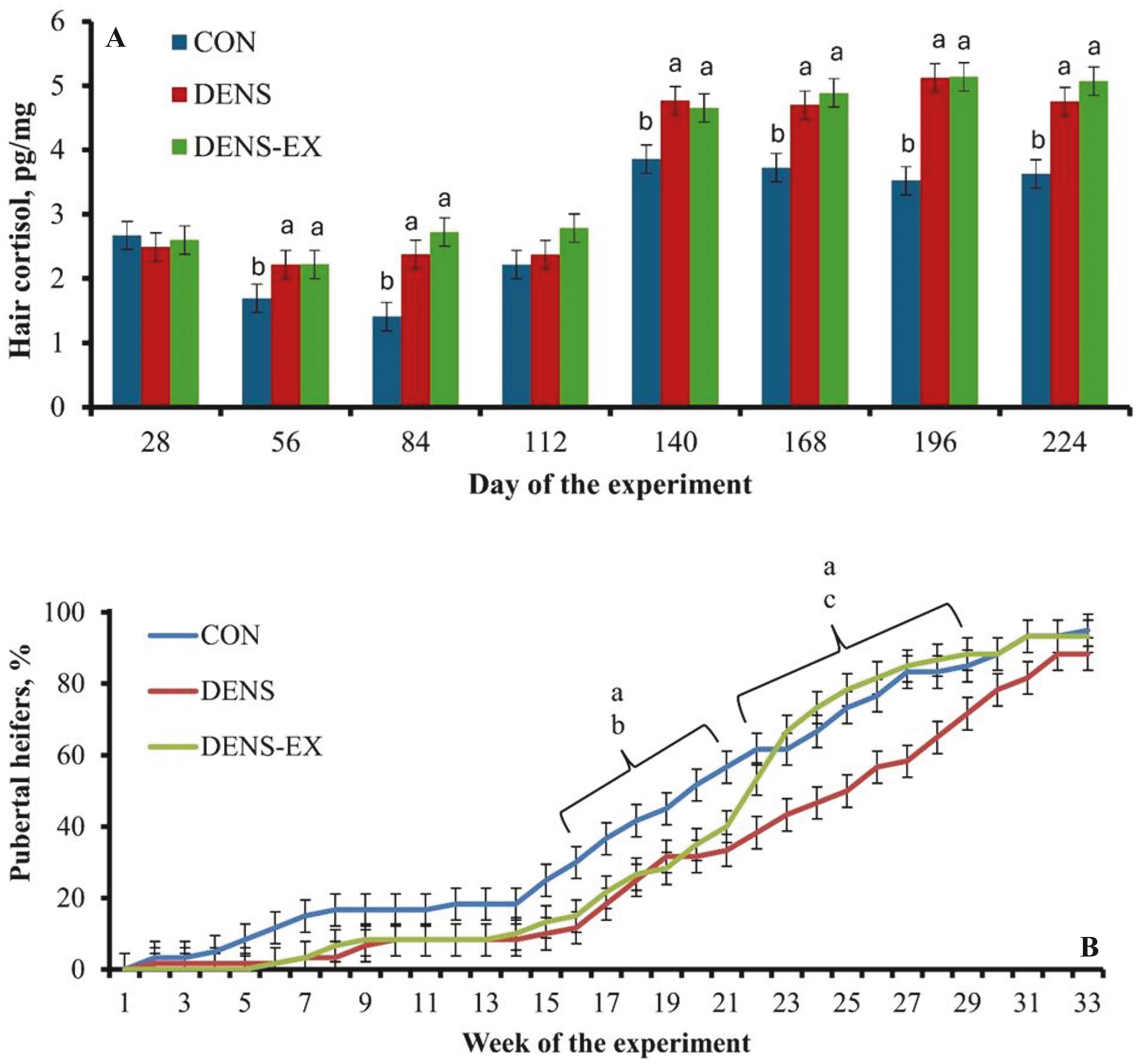
Hair cortisol concentrations (Panel A) and puberty attainment (Panel B) of replacement beef heifers reared in low stocking density (2,000 m^2^/heifer; **CON**), high stocking density (14 m^2^/heifer; **DENS**), or high stocking density (14 m^2^/heifer) with moderate exercise regimen (**DENS-EX**). Adapted from Harvey et al. (2024), and heifers averaged 264 days age on day 0. Treatment × day (or week) interactions were detected in both panels (*P* < 0.01). In Panel A, means with different superscripts differ (*P* ≤ 0.05). In Panel B, superscripts indicate treatment differences (*P* ≤ 0.05) between: a = CON vs DENS, b = CON vs. DENS-EX, c = DENS vs. DENS-EX.

Expression of natural behaviors in cattle, such as grazing and resting, indicates the environment and management conditions meet the species-specific needs, contributing to an optimal welfare state (Kilgour, 2012). This contrasts with more restrictive environments, such as DENS and DENS-EX heifers were housed in, where cattle were not able to express as much normal behavior and endured greater stress. Corroborating this rationale, hair cortisol concentration was greater for DENS and DENS-EX heifers for the majority of the experiment but did not differ between DENS and DENS-EX (Figure 4; Harvey et al., 2024). The results for hair cortisol indicate that chronic stress was greater for heifers reared in elevated stocking density compared with pasture reared cohorts, despite the exercise regimen applied to DENS-EX heifers.

Puberty attainment was delayed in DENS and DENS-EX heifers compared with CON heifers, whereas DENS-EX heifers reached puberty earlier compared with DENS heifers (Figure 4). Similarly to Schubach et al. (2017), age at puberty was less in CON vs. DENS heifers (391 vs. 417 days of age, respectively). Harvey et al. (2024) concluded that the greater chronic stress elicited by an elevated stocking density delayed puberty attainment in beef heifers. Implementing a moderate exercise regimen three times weekly did not fully counteract the physiological impacts of chronic stress evaluated, but it did partially alleviate the delay in puberty attainment compared with drylot reared heifers without exercise. Collectively, these results demonstrate that appropriate levels of physical activity positively influence puberty attainment and reproductive efficiency in beef females. Rearing heifers in intensive systems with a high stocking density restricts movement and limits the ability of heifers to engage in natural behaviors, resulting in chronic stress and delayed puberty attainment. Further research is warranted investigating exercise intensity and type on welfare and reproductive outcomes in heifers reared in drylot systems.

## Conclusions

Stress serves as a significant factor that can negatively impact replacement heifer growth, health, and future productivity. Heifer temperament and reproductive development are closely linked, with excitable heifers exhibiting delayed puberty attainment and conception to breeding compared with calmer cohorts. Acclimating heifers to human handling and management may be a strategy to lessen temperament-induced stress, thus favoring reproductive maturation. Additionally, rearing heifers in high stocking density, characterized by limited space per heifer, results in restricted movement, elevated chronic stress, and delayed puberty attainment. Moderate exercise regimen may be a strategy to alleviate the detrimental effects of high stocking density, highlighting that lack of exercise and freedom of movement directly contributes to delayed reproductive development of heifers reared in drylots. Therefore, temperament and exercise allowance should be considered to optimize the growth and reproductive development of beef heifers.

## About the Authors

**Kelsey M. Harvey** is an Assistant Professor at the Prairie Research Unit at Mississippi State University (MSU), where her research program focuses on nutritional and management strategies to optimize beef production efficiency. She received a BSc and MSc in Animal Science from Oregon State University, and PhD in Animal Science from Texas A&M University, where her work focused on heifer development and nutritional management during gestation and early life. Kelsey’s work at MSU focuses on optimizing cattle health, performance, and productivity through practical, science-based approaches to feeding and herd management. Her program has a specific emphasis on mineral nutrition in high-risk and mature cattle, and management of replacement beef heifers to optimize long-term productivity.

**Reinaldo Fernandes Cooke** serves as the Burkhart Endowed Professor for Beef Cattle Research in the Department of Animal Science at Texas A&M University. Dr Cooke received a BS (2003) in Animal Sciences from Sao Paulo State University (Brazil), and a MSc (2006) and a PhD (2008) in Animal Sciences from the University of Florida. Dr Cooke’s program is geared toward addressing the needs of the Texas, United States, and worldwide beef industries. His research efforts focus on management strategies to improve productive efficiency in beef cattle operations, including nutrition, health, growth, and reproductive responses in *Bos indicus* and *B. taurus* cattle. **Corresponding author:**reinaldocooke@tamu.edu

**Brooklyn L. Laubinger** holds a BSc in Animal and Dairy Sciences from Mississippi State University (MSU) and is currently pursuing her MSc in Animal Science from MSU. Her research focuses on the impact of postweaning nutritional regimens on the reproductive development and productivity of commercial beef heifers. Mrs. Laubinger is currently employed as a Research Associate for the North Mississippi Research and Extension Center-Prairie Research Unit where she helps maintain and contributes to ongoing research projects. She has a particular interest in management of gastrointestinal parasites in beef cattle, and utilizing alternative feed sources to reduce food waste and contribute to sustainable production efficiency in cattle.
